# Acute kidney injury following CAR-T cell therapy: a nephrologist’s perspective

**DOI:** 10.1093/ckj/sfae359

**Published:** 2024-11-15

**Authors:** Mehmet Kanbay, Berk Mizrak, Ezgi N Alper, Sidar Copur, Alberto Ortiz

**Affiliations:** Department of Medicine, Division of Nephrology, Koc University School of Medicine, Istanbul, Turkey; Department of Medicine, Koc University School of Medicine, Istanbul, Turkey; Department of Medicine, Koc University School of Medicine, Istanbul, Turkey; Department of Medicine, Koc University School of Medicine, Istanbul, Turkey; Department of Medicine, Universidad Autonoma de Madrid and IIS-Fundacion Jimenez Diaz, Madrid, Spain

**Keywords:** acute kidney injury, autoimmunity, chimeric antigen receptor T-cell therapy, nephrotoxicity, onconephrology

## Abstract

Chimeric antigen receptor T (CAR-T) cell therapy, an emerging personalized immunotherapy for various haematologic malignancies, autoimmune diseases and other conditions, involves the modification of patients’ T cells to express a chimeric antigen receptor that recognizes tumour or autoimmune cell antigens, allowing CAR-T cells to destroy cancerous and other target cells selectively. Despite remarkable clinical improvements in patients, multiple adverse effects have been associated with CAR-T cell therapy. Among the most recognized adverse effects are cytokine release syndrome, immune effector cell–associated neurotoxicity syndrome and tumour lysis syndrome. Even though less recognized, the incidence of acute kidney injury (AKI) ranges from 5 to 33%. The wide range of reported AKI incidence rates might depend on patient population characteristics and comorbidities and specific CAR-T cell therapy features. Even though the exact pathophysiology remains unknown, several key mechanisms, including cytokine release syndrome, tumour lysis syndrome and other factors such as direct renal toxicity of CAR-T cell therapy, conditioning regimens or other medications (e.g. antibiotics), and infectious complications (e.g. sepsis) have been proposed. Risk factors for CAR-T-related AKI include lower baseline glomerular filtration rate, higher rates of allopurinol or rasburicase use, intravenous contrast material exposure, elevated baseline lactate dehydrogenase and grade 3 or higher cytokine release syndrome. Future prospective studies with larger patient populations are needed to gain insights into the pathophysiology of CAR-T-related AKI and, more importantly, to be able to prevent as well as to develop novel and more efficient treatment modalities. In this narrative review, we discuss the underlying pathophysiology, risk factors, potential interventions and future directions related to AKI following CAR-T cell therapy.

## INTRODUCTION

Chimeric antigen receptor T (CAR-T) cell therapy has emerged as a revolutionary treatment for certain types of haematologic malignancies, particularly relapsed or refractory B cell acute lymphoblastic leukaemia (ALL) and diffuse large B cell lymphoma (DLBCL), as well as for autoimmune diseases and other conditions [1, 2]. This form of precision immunotherapy involves modifying a patient's T cells to express a CAR-T specific to cancer cells, thereby enhancing the immune system's ability to target and destroy cancerous cells specifically or other targets, such as B cells in systemic lupus erythematosus (SLE) [3, 4].

Following the demonstration of safety and efficacy, both the US Food and Drug Administration and the European Medicines Agency approved several CAR-T cell therapies such as tisagenlecleucel and axicabtagene ciloleucel for relapsed or refractory large B cell lymphoma [5, 6] and idecabtagene vicleucel and ciltacabtagene autoleucel for relapsed or refractory multiple myeloma [7, 8]. Clinical trials are ongoing or planned for conditions ranging from lupus nephritis (LN) to membranous nephropathy to solid tumours (e.g. NCT05085431, NCT06285279 and NCT06010875).

Despite its remarkable efficacy, CAR-T cell therapy is associated with a spectrum of adverse effects, including cytokine release syndrome (CRS), immune effector cell–associated neurotoxicity syndrome (ICANS), tumour lysis syndrome (TLS) and acute kidney injury (AKI) [9]. The incidence of AKI following CAR-T cell therapy varies across studies, with reported rates ranging from about 5% to 33% [10]. This variability depends on factors such as the patient population characteristics and comorbidities and the specific CAR-T cells involved [11]. This review discusses the pathophysiology, risk factors, potential interventions and future research directions for AKI related to CAR-T cell therapy.

### Pathophysiology of kidney injury

**Figure 1:**
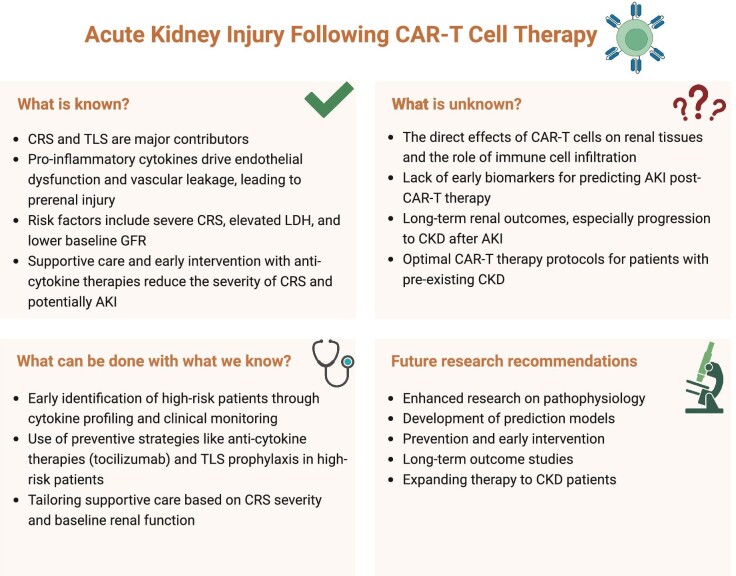
The knowns and unknowns regarding AKI following CAR-T cell therapy.

**Figure 2:**
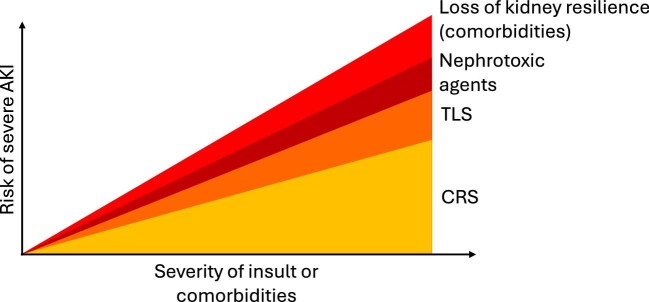
Factors contributing to the risk of severe AKI in relation to the severity of insult or comorbidities following CAR-T cell therapy.

The exact pathophysiology of AKI following CAR-T cell therapy is not entirely understood and is likely to be multifactorial. Several key mechanisms that contribute to the development of AKI have been proposed (Fig. 1) and their interaction determines the severity of AKI (Fig. 2).

#### CRS

CRS is an acute systemic inflammatory syndrome characterized by the presence of fever with or without accompanying hypotension, hypoxia or other end-organ dysfunction seen hours to days after the administration of either monoclonal antibodies, CAR-T cells or haploidentical haematopoietic stem cell transplantation [12]. Even though the exact underlying mechanism of CRS is unclear, the condition is associated with elevated levels of pro-inflammatory cytokines, including interleukin (IL)-6, interferon-gamma, tumour necrosis factor-alpha and granulocyte colony-stimulating factors [13]. CRS is among the most common complications of CAR-T cell therapy and is an important contributor to AKI development. Elevated levels of pro-inflammatory cytokines lead to vasodilatory response and capillary leakage syndrome, both of which predispose patients to pre-renal AKI via a reduction of effective intravascular volume and acute tubular necrosis if the process is severe or prolonged [14, 15]. Additionally, inflammatory cytokines have direct and indirect effects on kidney cells, as is well-characterized for kidney tubular cells. Thus they decrease the expression of kidney protective molecules such as Klotho, peroxisome proliferator-activated receptor gamma coactivator 1-α) and Nod-like receptor pyrin domain-containing protein 6 [16–18], promote a pro-inflammatory response, in part dependent on the expression of receptor-interacting protein kinase-3 by bone marrow cells [19], and may induce tubular cell death [20]. The cardiovascular effects of CRS, manifested as decreased cardiac output and cardiac index, may further exacerbate pre-renal AKI and acute tubular necrosis via cardiorenal syndrome [21]. Another contribution of pro-inflammatory cytokines is the endothelial dysfunction and injury to both podocytes and glomeruli along with a pro-coagulant state [22]. Endothelial activation and dysfunction are associated with elevated levels of multiple endothelial-related markers including von Willebrand factor, soluble intracellular adhesion molecule-1 and angiotensin-2 [23].

A serious and rare complication of CAR-T cell therapy, referred as haemophagocytic lymphohistiocytosis/macrophage activation syndrome, presents with findings similar to CRS despite slightly different underlying pathophysiology [24]. Although both conditions share the overactivation of the reticuloendothelial system induced by T cells, patients with CRS may not have hepatosplenomegaly, lymphadenopathy or evidence of haemophagocytosis in the liver, spleen or bone marrow. Nonetheless, most patients with moderate–severe CRS fulfil laboratory and clinical diagnostic criteria for haemophagocytic lymphohistiocytosis/macrophage activation syndrome [24]. Therefore, the differential diagnosis of these two conditions is not always straightforward, as 39 CAR-T cell–treated patients diagnosed with CRS also demonstrated histopathological evidence of haemophagocytic lymphohistiocytosis/macrophage activation syndrome [13]. The renal infiltration by activated cytotoxic T cells and macrophages may directly cause AKI of the renal type, i.e. with established kidney injury [25].

#### TLS

Haematological malignancies with rapid turnover and a high Ki-67 index are prone to develop TLS either spontaneously or induced by effective therapeutic regimens, the latter especially if the tumour bulk is large. A single-centre retrospective study involving 77 patients receiving either axicabtagene ciloleucel or brexucabtagene autoleucel observed low rates of patients meeting the criteria for laboratory or clinical TLS. The incidence of hyperuricaemia, hyperkalaemia and hyperphosphataemia were 3%, 1% and 14%, respectively, with only three patients demonstrating AKI [26]. Another clinical study investigating the incidence and risk factors for TLS in relapsed/refractory multiple myeloma treated with anti-B-cell maturation antigen CAR-T cell therapy reported a 17.1% incidence of TLS among 105 patients [27]. Patients with TLS were more likely to have higher levels of pro-inflammatory cytokines, including IL-6, interferon-gamma and ferritin levels. In this regard, all patients with TLS also had clinical signs of CRS, with 72.2% having grade 3–4 CRS. Moreover, TLS appears to be a negative indicator of long-term prognosis, including progression-free survival (*P* < .001) and overall survival (*P* < .001) [26]. Similar incidence rates have been reported in other clinical trials as well [28, 29]. Similar to TLS associated with conventional therapies, precipitation of calcium phosphate and uric acid crystals in renal tubules appears to be the primary mediator of TLS-mediated AKI with CAR-T cell therapy.

#### Other factors

Even though CRS and TLS are the most common mechanisms of kidney injury following CAR-T cell therapy, there are multiple other potential contributors as well. Such factors include direct renal toxicity of CAR-T cell therapy, such as collapsing glomerulopathy [30, 31], infectious complications including sepsis, renal toxicity of conditioning regimens, mostly including fludarabine and cyclophosphamide, and toxicity of other medications, including antibiotics. Fludarabine has been associated with low rates of nephrotoxicity (<5%) [32,33], similar to the low rates of nephrotoxicity associated with cyclophosphamide, except for haemorrhagic cystitis caused by acrolein, a metabolite of cyclophosphamide, and hyponatraemia caused by the syndrome of inappropriate secretion of antidiuretic hormone [34].

### Risk factors

**Figure 3:**
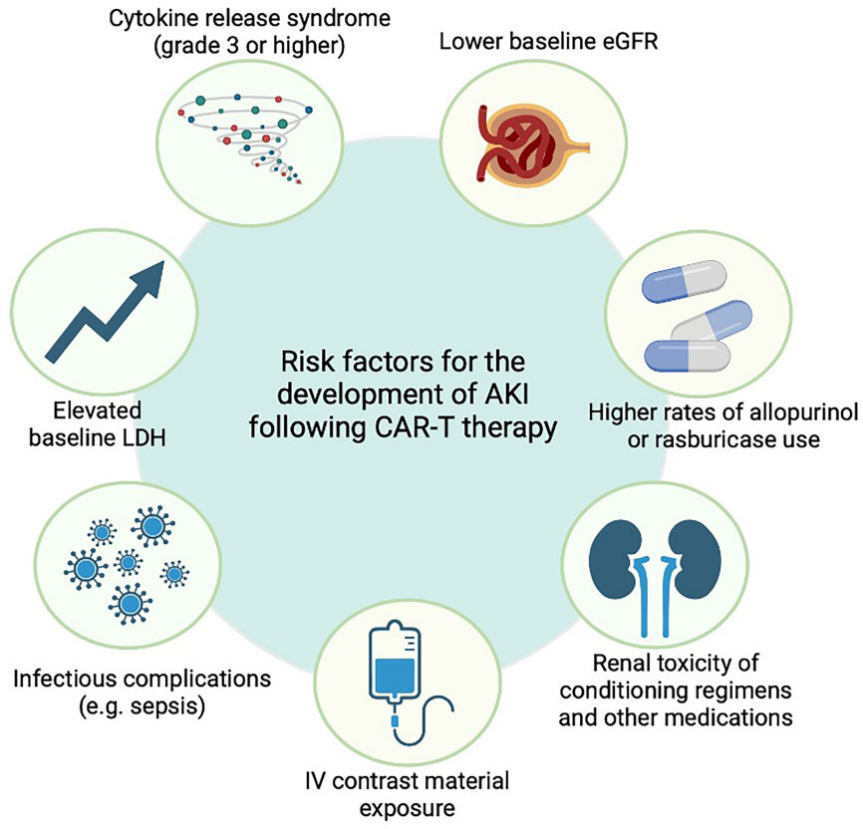
The potential risk factors for nephrotoxic adverse effects of CAR-T cell therapy.

As the understanding of the potential underlying mechanisms and epidemiology of CAR-T-related AKI increases, multiple patient- and therapy-related risk factors have been identified (Fig. 3). A single-centre retrospective analysis of 46 relapsed/refractory non-Hodgkin lymphoma (NHL) patients treated with either axicabtagene ciloleucel or tisagenlecleucel analysed risk factors for AKI. There was no statistically significant difference in the incidence of CRS (71% versus 81%) or ICANS (43% versus 47%) in AKI patients compared with no-AKI patients. However, AKI patients were more likely to experience grade 3–4 CRS (29% versus 6%) without differences in median ferritin or peak IL-6 levels. Thus the clinical severity of CRS appears to be a risk factor for AKI. Moreover, no differences were reported for median age, gender, pretreatment serum creatinine levels or prior lines of chemotherapy regimens [35]. Another single-centre retrospective observational study conducted on relapsed/refractory NHL patients identified lower baseline estimated glomerular filtration rate (eGFR) (80.4 versus 93.5 ml/min/1.73 m^2^; *P* = .047), higher rates of allopurinol or rasburicase use (71.4% versus 37.7%; *P* = .02) and intravenous contrast material exposure (64.3% versus 29.0%; *P* = .012) as risk factors for AKI. In this study, 14 of 83 patients developed AKI, mostly caused by intrinsic kidney damage (*n* = 10), with a median of 7.5 days after CAR-T cell therapy, and most AKI episodes resolved during follow-up (*n* = 10) [36]. Another study on 119 intensive care unit (ICU)-admitted patients after CAR-T cell therapy, of whom 34% developed AKI, identified elevated baseline lactate dehydrogenase (LDH) and, again, the severity of CRS (grade ≥3) as potential risk factors [37]. Identification of either elevated LDH levels or the need for tumour lysis prophylaxis as risk factors for AKI highlights the pathophysiological significance of TLS in AKI following CAR-T cell therapy.

#### Development of risk stratification and prediction models

Several studies have identified clinical factors associated with increased AKI risk, including high baseline LDH levels, the need for TLS prophylaxis and the severity of CRS. There is also emerging evidence that pre-existing chronic kidney disease (CKD) or lower baseline GFR may predispose patients to AKI. Certain therapeutic interventions, such as intravenous contrast use, have been linked to a higher AKI incidence after CAR-T cell therapy [38]. Despite these insights, no validated risk prediction models for AKI in CAR-T patients currently exist. The integration of cytokine profiles, renal biomarkers and patient-specific factors into a predictive framework is an area of active research as previously done for other validated AKI risk tools available online for other clinical scenarios. Furthermore, the potential impact of genetic predispositions or immunophenotypic variations in influencing AKI risk has yet to be explored.

### Epidemiology and outcomes

**Figure 4:**
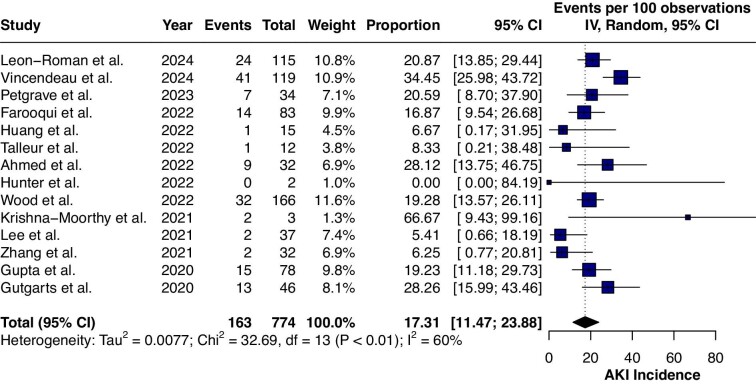
The analysis of clinical studies evaluating the incidence of AKI following CAR-T cell therapy.

Multiple clinical studies have and are evaluating the incidence and risk factors for AKI following CAR-T cell therapy. Our literature search identified 14 clinical studies evaluating the risk factors and incidence of AKI following CAR-T cell therapy. Overall, 163 AKI episodes were observed among 774 patients [17.3% (95% CI 11.47–23.88)] (Fig. 4). Clinical studies are summarized in Table 1. Studies that have data for patients with pre-existing kidney disease are summarized in Table 2. A single-centre retrospective study reported on 119 patients admitted to the ICU after CAR-T cell therapy out of 377 patients receiving CAR-T cell therapy [37]. The mean age was 55 years and the incidence of AKI (*n* = 41) among those admitted to the ICU was 34%, likely an overestimation of the incidence of AKI following CAR-T cell therapy. Additionally, the study included patients with at least three previous lines of chemotherapy. The most common complications leading to ICU admission include grade 2–3 CRS or sepsis from bacterial infections. AKI following CAR-T cell therapy was mostly Kidney Disease: Improving Global Outcomes (KDIGO) stage 1 (61%), while 7% (*n* = 3) required kidney replacement therapies [37]. A large-scale retrospective clinical study involving a total of 115 adult patients with relapsed/refractory haematological malignancies reported an incidence of AKI of 21% (*n* = 24), with rapid recovery of renal functions in 79% of the patients (*n* = 19) within a 1-month period [39]. Similarly, other retrospective clinical studies have reported low rates of AKI along with rapid recovery in most cases [36, 40].

**Table 1:** The major clinical studies evaluating the potential nephrotoxic adverse effects of CAR-T cell therapy.

Author	Year	Patients, *N*	Sex	Indication for CAR-T therapy	CAR-T treatment choice	AKI incidence, *n* (%)	AKI stage/definition	CRS incidence, *n* (%)	CRS grade (%)	Potential underlying cause for AKI	Outcome (mortality and morbidity)
Farooqui *et al.* [36]	2022	83	Male 65.1%	Aggressive NHL	Anti-CD 19 CAR-T cells (axicabtagene ciloleucel)	14 (17)	KDIGO stage 1: 11 (78.5), stage 2: 1 (7.2), stage 3: 2 (14.3)	71 (85.5), both AKI and CRS: 12 (14.45)	AKI: grade 0: 2 (14.3), grade 1: 5 (35.7), grade 2: 6 (42.9), grade 3: 0 (0), grade 4: 1 (7.1)	Lower baseline eGFR, use of IV contrast, tumour lysis prophylaxis, higher peak uric acid and CK, elevated LDH, higher doses of CSs and tocilizumab CRS	At 1 month post-CAR-T, 71% of AKI patients had resolved. Among 14 patients with AKI, 4 died during the 6-month follow-up; 1 patient requiring RRT died within 48 hours due to severe CRS
Gupta *et al.* [40]	2020	78	Male 64%	DLBCL	Axicabtagene ciloleucel (88%), tisagenlecleucel (12%)	15 (19)	KDIGO	66 (85)	Grade 0: 12 (15), grade 1: 28 (36), grade 2: 28 (36), grade 3: 8 (10), grade 4: 2 (3)	CRS, decreased kidney perfusion, acute tubular necrosis, urinary obstruction	Among 15 AKI patients, 8 had decreased kidney perfusion, 6 developed ATN and 1 had urinary obstruction. Those with ATN/obstruction had the longest hospital stay and highest 60-day mortality
Gutgarts *et al.* [35]	2020	46	Male 72%	DLBCL	Axicabtagene ciloleucel (74%), tisagenlecleucel (26%)	13 (30)	KDIGO stage 1: 21.7%, stage 2–3: 8.7%	37 (80)	AKI: grade 1–2: 24 (75), grade 3–4: 2 (6)	CRS IV contrast administration, tumour lysis syndrome	3 patients in the AKI group died within 3 weeks. Of the remaining 11, 10 had reversed kidney function within 30 days, except 1 patient who had a creatinine level 1.5 times higher than baseline
Huang *et al*. [53]	2022	15	NA	B-ALL involving extramedullary relapse	CD19 CAR-T cell therapy	1 (6.7)	NA	13 (86.7)	Grade 1–2: 7 (53.8), grade 3: 6 (46.2)	NA	NA
Krishna-Moorthy *et al.* [48]	2021	3	Male 66%	Refractory and/or relapsing PTLD with histology of DLBCL	Axicabtagene ciloleucel (Axi-cel)	3 (100)	NA	3 (100)	Patient 1: grade 1, patient 2: grade 2, patient 3: grade 3	Decreased effective circulating volume due to hypotension from CRS leading to ATN	Patient 1: Transition to hospice due to refractory PTLD with death at day 115, patient 2: transition to hospice with death at day 44 due to persistent lower GI bleed from refractory PTLD, patient 3: terminally extubated to comfort care, with death at day 15 from with refractory PTLD
Lee *et al.* [54]	2021	37	Male 65%	DLBCL	Tisagenlecleucel	2 (5)	Stage 3 AKI: 2 (100)	20 (54)	Grade 1: 11, grade 2: 4, grade 5: 1	Cytokine-mediated vasodilation and capillary leak MAS/HLH	Lower rates of AKI (5%) in patients receiving tisagenlecleucel compared with prior series evaluating AKI after CAR-T. Lower rates of hyponatraemia and hypokalaemia after tisagenlecleucel, which may also be due to lower rates of CRS. However, severe hypophosphataemia <2.0 mg/dl was extremely high in both this and a prior series, affecting ≥70% of patients. Identified the first case of new-onset proteinuria and AKI occurring in a patient with CAR-T-triggered MAS/HLH
Leon-Roman *et al.* [39]	2024	115	Male 66%	Diffuse large B cell lymphoma (91%), B cell acute lymphoblastic leukaemia (5%), mantle cell lymphoma (4%), primary mediastinal large B cell lymphoma (1%)	Lisocabtagene maraleucel, tisagenlecleucel, axicabtagene ciloleucel, brexucabtagene autoleucel	24 (21)	KDIGO stage 1: 17 (14.8), stage 2: 4 (3.5), stage 3: 3 (2.6)	95 (82)	AKI: grade ≥2: 15 (71.4)	Male gender, CKD, CRA, ICANS	6/24 patients that had developed AKI related to CAR-T therapy had CKD. Renal function was recovered in 19/24 patients (79%) within the first month after CAR-T infusion
Petgrave *et al.* [55]	2023	34	Male 55.9%	Relapsed and/or refractory CD19-positive malignancy	Tisagenlecleucel or an institutional academic product (NCT03573700)	7 (20.6)	KDIGO stage 2–3: 4 (57.1)	21 (60)	AKI: grade 0: 0 (0), grade 1–2: 3 (20), grade 3–4: 66.7 (4)	CRS, neurotoxicity	Among the 7 patients with AKI, 5 recovered kidney function and 2 died prior to recovery of kidney function
Talleur *et al.* [56]	2022	12	Male 33.3%	Relapsed/refractory B-ALL	CD19 CAR-T cells	1 (8.3)	Grade 3 (100)	6 (50)	Grade 3–4: 2 (33.3)	CarHLH	NA
Vincendeau *et al.* [37]	2024	119	Male 62%	Diffuse large B cell lymphoma: 93 (75.6), ALL: 22 (22), myeloma: 3 (2.5)	Brexucabtagene autoleuce (Tecartus), tisagenlecleucel (Kymriah), axicabtagene ciloleucel (Yescarta), other	41 (34)	KDIGO stage 1: 25 (61). stage 2: 6 (14.6), stage 3 10 (24.4)	Isolated CRS: 22 (18.6), CRS: 109 (91.6), AKI: 36 (30.3)	AKI: grade 0: 4 (9.8), grade 1: 7 (17.1), grade 2: 15 (36.6), grade 3: 14 (34.1), grade 4: 1 (2.4)	CRS, elevated LDH levels	82% of survivors had kidney recovery at discharge. 9/12 (75%) and 6/9 (67%) with AKI and survived had CKD at 56 months and 1 year, respectively. Mortality was higher in AKI KDIGO 2 or 3 group
Zhang *et al.* [57]	2021	32	Male 59.4%	R/R B-NHL	CD19/22 CAR-T cells	2 (6.3)	Stage 4: 1 (3.1), stage 5: 1 (3.1)	29 (90.6)	Grade 1: 14 (43.18), grade 2: 6 (18.7), g rade 3: 5 (15.6), grade 4: 3 (9.4), grade 5: 1 (3.1)	CRS	1 patient died due to severe CRS-associated AKI

ALL: acute lymphoblastic leukaemia; CarHLH: CAR-T cell therapy mediated hemophagocytic lymphohisticytosis; DLBCL: diffuse large B cell lymphoma; CK: creatinine kinase; ATN: acute tubular necrosis; IV: intravenous; NA: not available.

**Table 2:** Safety and outcomes in patients with pre-existing kidney disease, reduced renal function and/or requirement of haemodialysis.

Author	Year	Patients, *N*	Sex	Indication for CAR-T therapy	CAR-T treatment choice	AKI incidence, *n* (%)	AKI stage/definition, *n* (%)	CRS incidence, *n* (%)	CRS grade (%)	Potential underlying cause for AKI	Outcome (mortality and morbidity)
Ahmed *et al.* [49]	2022	32 (total), 7 pre-existing CKD, 25 without CKD	Male 56%	DLBL 94%, MCL 6%	Tisagenlecleucel 12%, brexucabtagene autoleucel 6.2%, axicabtagene ciloleucel 81%	9 (28): no CKD: 7 (28), yes CKD: 2 (29)	KDIGO No CKD: grade 1: 2 (29), grade 2: 3 (43), grade 3: 2 (29), grade 4–5: 0 Yes CKD: grade 1: 2 (100), grade 2: 0, grade 3: 0, G4–5: 0	No CKD: 25 (88), no AKI: 23 (100), yes CKD: 7 (100), yes AKI: 9 (100)	No AKI (*N* = 23): grade 0–1: 13, grade ≥2: 10 Yes AKI (*N* = 9): grade 0–1: 7, grade ≥2: 2	Serum creatinine at baseline ICANS grade 2+	18/32 patients died at the last follow-up; however, renal failure was not the primary cause of death in any of the patients
Hunter *et al.* [52]	2022	2	Both female	DLBCL ESRD	Axicabtagene ciloleucel, lisocabtagene maraleucel	0	NA	1 (50)	Grade 1: 1 (100)	NA	None experienced AKI; however, the first patient died due to non-kidney-related health problems 4 months after CAR-T therapy
Wood *et al.* [50]	2022	166 (total): baseline RI: 17 (10.2%), without RI: 149 (89.8)	Male 56%	DLBCL	Axicabtagene ciloleucel, tisagenlecleucel	Baseline RI: 7/17 (42%), non- RI: 32/149 (21%)	KDIGO RI: grade 2–3: 1/17 (6), non-RI: grade 2–3: 11/49 (7)	AKI group: 17/39 (44)	RI: grade ≥3: 2/17 (12), non-RI: grade ≥3: 12/149 (8); AKI during CART: yes: 9/39 (23), no: 5/127 (4)	Decreased renal perfusion: 28/39 (72%), CRS: 17/39 (44%), intrinsic renal injury: 10/39 (26%)	The incidence of AKI was similar between patients with and without baseline renal impairment (RI) (42% versus 21%; *P* = .08). PFS and OS were comparable across RI status and fludarabine dosing, however, patients with AKI had significantly worse outcomes [PFS HR 2.1 (95% CI 1.2–3.7); OS HR 3.9 (95% CI 2.1–7.4)]. Renal recovery by day 30 post-AKI was similar in the RI and non-RI groups (71% versus 69%; *P* = 1). Peak inflammatory cytokines were higher in AKI patients

DLBCL: diffuse large B-cell lymphoma; MCL: mantle cell lymphoma; ESRD: end-stage renal disease; RI: renal impairment; PFS; progression-free survival; OS: overall survival; NA: not available.

### CAR-T to treat kidney diseases

The promising results of CAR-T cell therapy in the management of various haematological malignancies have raised hopes for their use in benign conditions as well. Even though research is limited, the risk of developing kidney injury with CAR-T cell therapy use in autoimmune conditions compared with haematological malignancies with high tumour burden is lower based on current knowledge; as the development of complications, such as CRS, is known to be more likely in cancer patients. Among the autoimmune diseases, SLE is a complex and heterogeneous autoimmune condition with variable clinical manifestations primarily caused by immune-complex deposition. CAR-T cell therapy has emerged as a promising therapeutic alternative for patients with refractory lupus, although clinical experience regarding such a therapeutic approach is very limited. A notable case report for the successful use of CD19-targeted CAR-T cell therapy for refractory lupus was published in 2021. A 20-year-old female with active lupus disease (i.e. nephritis, serositis, mucocutaneous and musculoskeletal involvement) that had been refractory to high-dose corticosteroids, multiple conventional immunosuppressive agents, rituximab and belimumab therapy over the years received CAR-T cell therapy that led to rapid remission within 44 days, with normalization of complement levels, negativization of anti-double-strand DNA antibodies and a decrease in proteinuria. Remission has been maintained without signs of flare-ups for 18 months with only low-dose prednisolone maintenance therapy [41]. The same authors later reported a case series comprised of five refractory cases of active lupus disease that were successfully treated with CAR-T cell therapy without any disease flare-up or need for maintenance immunosuppression during follow-up [3].

Another study showed that CAR-T treatment can eliminate autoantibodies, resets the immune system and provides long-term, medication-free remission in patients with SLE and LN. In addition, CAR-T treatment was well-tolerated, with no severe adverse events, and led to significant improvements in renal function in LN patients within 6 months [42].

Similar beneficial therapeutic effects were reported in another clinical trial enrolling seven refractory cases of SLE with a median follow-up period of 13 months [43]. These clinical studies investigating the efficiency of CD19-targeted CAR-T cell therapy on refractory cases of SLE shared a similar conditioning regimen comprised of fludarabine and cyclophosphamide and administered 1–1.1 × 106 autologous CAR-T cells/kg body weight. Adverse effects were limited to variable grades of CRS. Multiple ongoing clinical trials (NCT05869955, NCT05930314, NCT05858684, NCT05846347, NCT05765006, NCT05474885, NCT05030779 and NCT05085418) are evaluating the efficiency and safety of CAR-T cell therapy in SLE patients.

Even though the major source of information on CAR-T cell therapy and rheumatological disorders is studies on SLE patients, CAR-T cell therapy is rapidly evolving beyond SLE. A few cases indicating the successful and safe use of CAR-T cell therapy on systemic sclerosis and anti-synthetase syndrome patients have been reported with similar conditioning regimens and doses of anti-CD19 CAR-T therapies [44–46], along with multiple ongoing clinical trials (NCT05085431, NCT05085444 and NCT05859997).

### Special populations

The clinical experience of and knowledge regarding CAR-T cell therapy on prior solid organ transplant recipients is highly limited, as most such patients have been excluded from clinical trials due to their concurrent use of immunosuppressive medications. Nonetheless, despite the limited knowledge and experience in this area, several case reports and series offer significant insights and findings. A case report has described tubulointerstitial nephritis as a cause of kidney injury in a patient following CAR-T cell therapy for post-transplant lymphoproliferative disorder [47]. Furthermore, a case series involving three solid organ transplant recipients undergoing CAR-T cell therapy for immunochemotherapy-refractory post-transplant lymphoproliferative disorder illustrated the challenges in this scenario. This case series includes a kidney and pancreas transplant recipient with type 1 diabetes mellitus, a heart transplant recipient and a kidney transplant recipient. All patients were Epstein–Barr virus negative and developed post-transplant lymphoproliferative disorder at least 10 years after transplantation and received at least two prior lines of chemotherapy. All patients experienced high-grade CRS requiring tocilizumab therapy, while two patients developed AKI requiring renal replacement therapy. None of the patients survived, as they developed multiple therapy-related complications, indicating the low success rate of CAR-T cell therapy in solid organ transplant recipients [48]. Even though future preclinical and clinical studies are required for a better understanding of such issues, CAR-T cell therapy appears to be associated with significant therapy-related morbidity and mortality when considering potential drug interactions, infectious complications and CRS.

Following the discussion on transplant patients, CKD emerges as another special population at heightened risk for AKI, warranting an exploration of the efficacy and safety of CAR-T cell therapy. A single-centre retrospective clinical study involving a total of 32 patients—7 patients with pre-existing CKD— showed no significant effect of baseline CKD status on outcomes of CAR-T cell therapy in terms of overall survival or progression-free survival [49]. Similarly, another single-centre retrospective clinical study involving a total of 166 patients with relapsed refractory NHL—17 patients with baseline CKD—demonstrated no difference in terms of AKI incidence, grade 2/3 AKI incidence, overall survival or progression-free survival in patients with baseline CKD or not [50]. A systematic review and meta-analysis study included 252 patients from nine clinical studies investigating the efficiency and safety of CAR-T cell therapy on patients with pre-existing AKI or CKD. The median age was 48 years (range 19–86), with the most common primary diagnosis being NHL (*n* = 129) and diffuse large B cell lymphoma (*n* = 120). CAR-T cell therapy was highly effective even for patients with kidney failure irrespective of the pre-existing kidney disease status [9]. Moreover, a few case reports have indicated the successful use of CAR-T cell therapy among CKD patients, even among patients on maintenance haemodialysis [51, 52]. To conclude, CAR-T cell therapy appears to be a safe and effective therapeutic modality for patients with baseline CKD despite the clear need for future large-scale prospective clinical trials.

## CONCLUSION

CAR-T cell therapy is a revolutionary treatment for the management of multiple haematologic, rheumatologic and nephrologic disorders, though such revolutionary therapy is not without significant complications, including CRS and neurotoxicity. As CAR-T cell therapy is a novel concept, the knowledge regarding potential nephrotoxic adverse effects is limited. However, a high risk of AKI has been reported, likely resulting from a combination of CRS, TLS, nephrotoxic medications, sepsis and tubulopathies. Ongoing and future clinical trials will further inform the epidemiology of AKI related to CAR-T cell therapy in diverse clinical contexts and for diverse CAR-T approaches. Additionally, preclinical studies evaluating underlying pathophysiological mechanisms may inform prevention and therapeutic strategies.
